# Clinical feasibility of deep learning-based auto-segmentation of target volumes and organs-at-risk in breast cancer patients after breast-conserving surgery

**DOI:** 10.1186/s13014-021-01771-z

**Published:** 2021-02-25

**Authors:** Seung Yeun Chung, Jee Suk Chang, Min Seo Choi, Yongjin Chang, Byong Su Choi, Jaehee Chun, Ki Chang Keum, Jin Sung Kim, Yong Bae Kim

**Affiliations:** 1 Department of Radiation Oncology, Yonsei Cancer Center, Yonsei University College of Medicine, 50-1 Yonsei-ro, Seodaemun-gu, Seoul, 03722 Korea; 2 Department of Radiation Oncology, Ajou University School of Medicine, Suwon, Korea; 3 CorelineSoft, Co, Ltd, Seoul, Korea

**Keywords:** Breast cancer, Auto-segmentation, Deep learning, Clinical target volume, Organs-at-risk

## Abstract

**Background:**

In breast cancer patients receiving radiotherapy (RT), accurate target delineation and reduction of radiation doses to the nearby normal organs is important. However, manual clinical target volume (CTV) and organs-at-risk (OARs) segmentation for treatment planning increases physicians’ workload and inter-physician variability considerably. In this study, we evaluated the potential benefits of deep learning-based auto-segmented contours by comparing them to manually delineated contours for breast cancer patients.

**Methods:**

CTVs for bilateral breasts, regional lymph nodes, and OARs (including the heart, lungs, esophagus, spinal cord, and thyroid) were manually delineated on planning computed tomography scans of 111 breast cancer patients who received breast-conserving surgery. Subsequently, a two-stage convolutional neural network algorithm was used. Quantitative metrics, including the Dice similarity coefficient (DSC) and 95% Hausdorff distance, and qualitative scoring by two panels from 10 institutions were used for analysis. Inter-observer variability and delineation time were assessed; furthermore, dose-volume histograms and dosimetric parameters were also analyzed using another set of patient data.

**Results:**

The correlation between the auto-segmented and manual contours was acceptable for OARs, with a mean DSC higher than 0.80 for all OARs. In addition, the CTVs showed favorable results, with mean DSCs higher than 0.70 for all breast and regional lymph node CTVs. Furthermore, qualitative subjective scoring showed that the results were acceptable for all CTVs and OARs, with a median score of at least 8 (possible range: 0–10) for (1) the differences between manual and auto-segmented contours and (2) the extent to which auto-segmentation would assist physicians in clinical practice. The differences in dosimetric parameters between the auto-segmented and manual contours were minimal.

**Conclusions:**

The feasibility of deep learning-based auto-segmentation in breast RT planning was demonstrated. Although deep learning-based auto-segmentation cannot be a substitute for radiation oncologists, it is a useful tool with excellent potential in assisting radiation oncologists in the future.

*Trial registration* Retrospectively registered.

## Background

Modern radiotherapy (RT) planning is a complex process that relies on computed tomography (CT)-based three-dimensional (3D) imaging as well as an expert team [1]. Based on CT simulations, radiation oncologists contour the relevant target volumes and surrounding normal structures and communicate with the dosimetrist the anticipated dosimetric goals that will deliver a therapeutic radiation dose to the target while sparing the organs-at-risk (OARs). In contrast to other primary malignancies such as lung and head & neck cancer, modern RT planning has not been not commonly applied to breast cancer, in which conventional formulaic field-based planning and two-dimensional techniques have been predominantly used [2].

Recently, as the use of comprehensive regional node RT is increasingly being supported by multiple landmark trials [3–6], the complexity of the target volume has also increased, and international guidelines for regional node irradiation have been developed [7, 8]. Although RT for breast cancer patients is known for its low rates of acute and late toxicity [9–11], studies have demonstrated that incidental doses to the contralateral breast, esophagus, thyroid, and axillary-lateral thoracic vessel junction can affect patients’ quality-of-life [12–14]. Furthermore, some studies have suggested that radiation-induced damage to the lung and heart can even offset the benefit of loco-regional breast cancer RT [15]. However, quality issues and inter-physician variations of target volumes and OAR contours have been of particular concern arising from dummy runs, multi-institutional studies, individual case reviews and audit studies [16–19]. Uncertainties regarding volume delineation and subsequent target and normal tissue doses may not only decrease the treatment efficacy but also increase the complication risk.

With recent advances in computing power, algorithms, and data collection, artificial intelligence (AI) is increasingly being used in healthcare to assist physicians. In radiation oncology, there are numerous areas in which AI is applicable, such as target and normal tissue segmentation, dose optimization, decision support systems, application of predictive models, and quality assurance [20–22]. Auto-contouring tools have been adopted by an increasing number of physicians and have resulted in improved efficiency, particularly for OARs in head and neck cancer and target volume in prostate cancer [23, 24]. As there is a paucity of data regarding the auto-segmentation of target volumes and OARs in breast RT planning, we attempted to train a deep learning-based auto-segmentation model for target volumes and OARs for breast cancer and evaluated its clinical utility from a clinician’s perspective.

## Methods

### Patients

The study was approved by the institutional review board of Severance hospital (IRB: 4–2019-0339). It included 111 breast cancer patients who received adjuvant RT after breast-conserving surgery (BCS). Both left-sided and right-sided breast cancer patients were included. The median age of the patients was 51 years (range 28–77 years), and the median body mass index was 22.5 kg/m^2^ (range 17.03–35.4 kg/m^2^). For T stage, 15 patients were Tis (14%), 60 patients were T1 (54%), 33 patients were T2 (30%), and 3 patients were T3 (3%). For N stage, 82 patients were N0 (74%), 26 patients were N1 (23%), and 3 patients were N2 (3%). RT field included whole breast (WB) only for 79 patients (71%), whereas it included the WB with regional lymph nodes for 32 patients (29%). Both non-contrast (n = 50) and contrast-enhanced (n = 61) planning CT scans were used for manual delineation of CTVs and OARs. Planning CT scan (Somatom Sensation Open syngo CT 2009E, Siemens and Aquilion TSX-201A, Toshiba) was performed approximately two weeks prior to RT with a CT slice thickness of 3 mm. The setup position for all planning CT scans was the supine position with both arms held up using an arm support device (CIVICO). Contrast-enhanced planning CT was performed 1 min after administration of 80–90 mL intravenous contrast (iohexol, 84.11 g / 130 mL; depending on the patient’s weight).

### Delineation

Previous contours used for patient treatment were not used in this study. For homogeneity, a single expert who is ESTRO teaching course certified and treats approximately 550 breast cancer patients per year contoured the CTVs and OARs within 1 month, with the patients’ clinical information blinded. The target volume consisted of CTVs of right and left breasts (CTVp_breast); axillary levels 1, 2, and 3 (CTVn_L1, L2, L3); internal mammary chain (CTVn_IMN); and lymph node level 4 (CTVn_L4), which is supraclavicular lymph node delineated according to the ESTRO guidelines [7]. In our study, we included interpectoral nodes mentioned in the ESTRO guidelines in CTVn_L2. In addition, the supraclavicular lymph nodes were additionally delineated according to the RTOG guidelines (CTVn_SCL RTOG) [25]. The OARs included the heart, right and left lungs, esophagus, spinal cord, and thyroid [26].

### Deep learning-based auto-segmentation

**Fig. 1:**
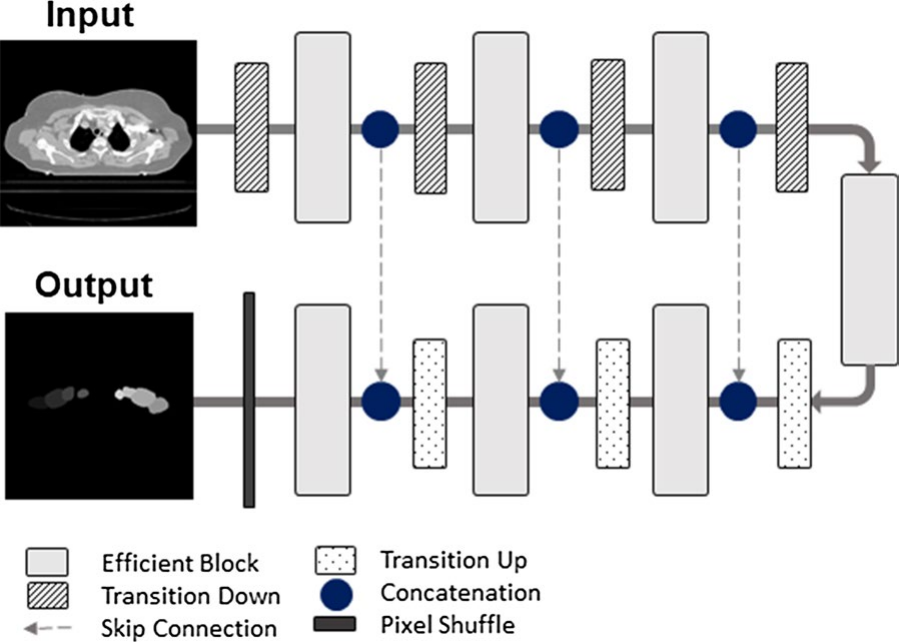
Schematic of the proposed convolutional neural network architecture (U-Net with EfficientNet-B0)

To segment the CTVs and OARs, a 3D U-Net-like convolutional neural network (CNN) was used, which was based on U-Net structure [27], and combined with 3D version of EfficientNet-B0 as the backbone (Fig. 1) [28]. In the CNN, instead of using 2D operations as in U-Net and EfficientNet, their 3D counterparts are used to exploit the 3D structural information. For inputs of the CNN, all cases were resampled to a voxel spacing of 1.0 × 1.0 × 3.0 mm3 and then the image intensity values of a truncated range of [-160, 240] were linearly normalized into the range of [0, 1]. Owing to GPU memory limitations, the CNN was trained in the patch level, specifically, input the 3D patch with a size of 128 × 128 × 64 from the volumetric CT images and output the 3D patch of multi-label segmentation. Furthermore, we trained the CNN with the sum of cross-entropy and dice loss, and set the optimizer to be RMSprop with an initial learning rate of 5 × 10–4. During training, in order to reduce overfitting and improve generalization, we employed regularization of weight decay of 10–4 [29], and data augmentation techniques such as scaling, flip, and rotation to all training patches.

### Analysis

Among the 111 cases that were newly contoured by an expert, 92 cases were used as the training dataset, and 19 cases were used as test dataset #1 (contrast: 10 cases; non-contrast: 9 cases) for the analysis of the quantitative metrics. Test dataset #2 was prepared separately to analyze the efficacy of auto-segmented contours using real-world heterogeneous data. Dosimetric parameters were analyzed using different sets of CT scans with manual contours (previously used for patient treatment) delineated by various physicians and RT plans of breast cancer patients who received RT after surgery (n = 42).

Both quantitative metrics and qualitative scoring were used for analyzing test dataset #1. Quantitative metrics included the most commonly used geometrical indices, such as Dice similarity coefficient (DSC) and 95% Hausdorff distance (HD), to compare the auto-segmented and manually delineated contours. DSC is a measure of overlap between two contours, from “0” to “1,” where “1” indicates a complete overlap. HD is the measure of distance between two contours, where 0 mm indicates a complete overlap. For qualitative scoring, two panels—an expert breast cancer radiation oncologist panel (n = 11) and a non-expert panel that included residents and radiation oncologists whose specialty is not breast cancer (n = 15)—from 10 institutions answered the following questions after watching an example video on manual contouring and auto-segmentation contouring on a planning CT scan:

1. What score would you give for the differences between manually delineated contours and auto-segmentation contours? (*Difference scores*)
  i. Answer: 0 (most different) to 10 (least different)
2. How much do you think auto-segmentation would assist you in real-world clinical practice? (*Assistance scores*)
  i. Answer: 0 (not helpful) to 10 (very helpful)

To analyze test dataset #2, auto-segmented contours were generated in 42 patients’ CT scans, and dose-volume histograms were analyzed using both auto-segmented contours and original manual contours. Furthermore, dosimetric analysis was performed by comparing the mean dose (Gy), D_0.03cc_ (Gy), and V_5Gy_ (cc) for heart; mean dose (Gy), V_20Gy,_ (%), and V_5Gy_ (%) for ipsilateral lung; mean dose for contralateral lung; D_0.03cc_ (Gy) for esophagus; and D_1cc_ (Gy) for spinal cord for the manual and auto-segmented contours.

Additionally, inter-user variability was assessed by analyzing the DSCs and HDs of contours delineated by three different radiation oncologists on a randomly selected CT scan of a breast cancer patient. Furthermore, the contouring time was recorded for all three radiation oncologists to compare the time taken for manual delineation with that for auto-segmentation. These results for inter-user variability are included as Additional file 1.

## Results

### Quantitative metrics

**Fig. 2:**
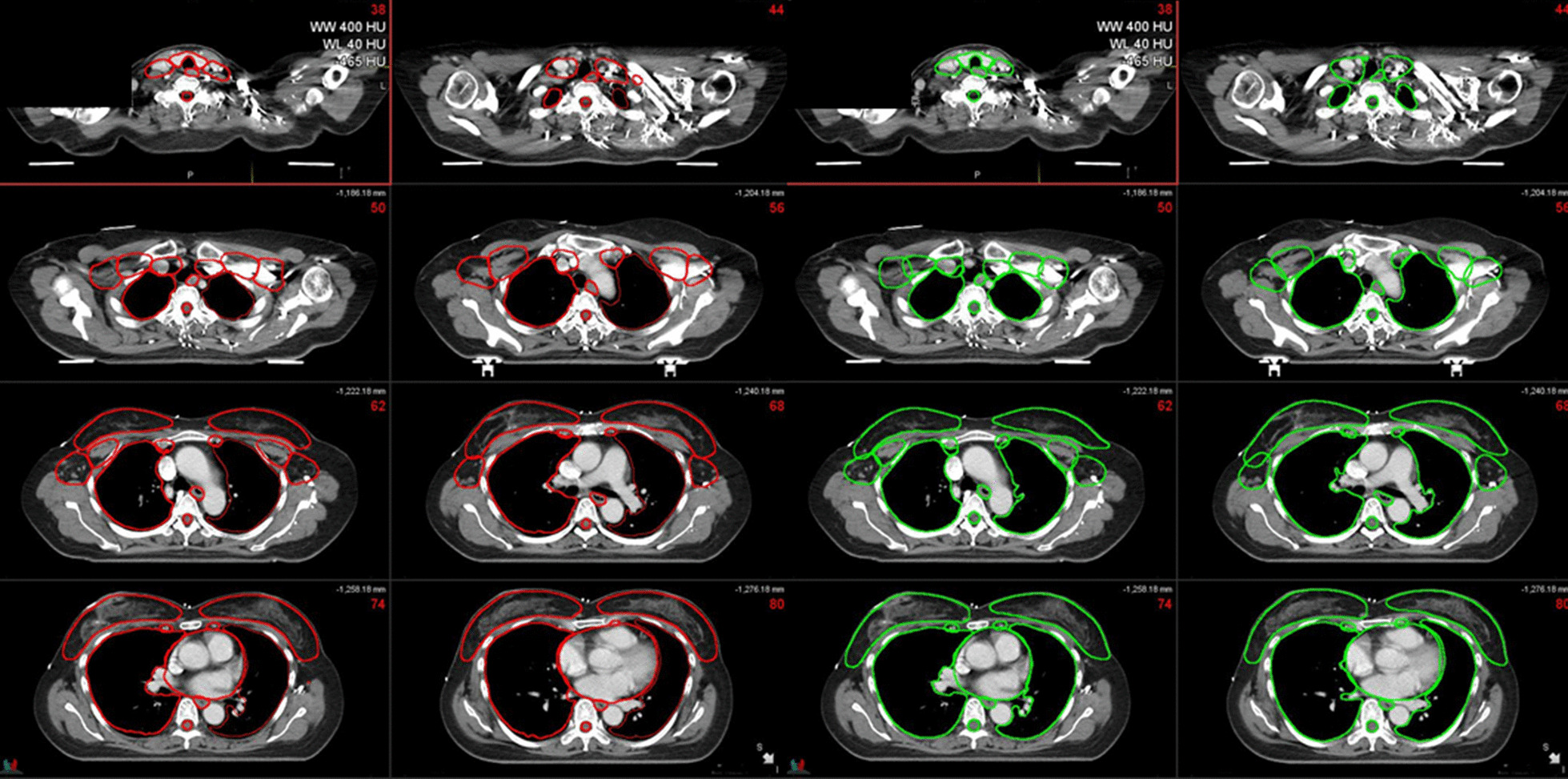
Example of deep learning-based auto-segmentation (green) and manual (red) contours

Examples of deep learning-based auto-segmentation and manual contours are shown in Fig. 2 and as a video in Additional file 2. Table 1 compares the auto-segmented contours and manual contours for OARs and CTVs using mean DSC and 95% HD. With regard to OARs, the mean DSCs were above 0.80, and mean 95% HDs were below 5 mm, which are acceptable results. For CTV, the correlation between the auto-segmented and manual contours was excellent for the breast, with a mean DSC higher than 0.90. As for other CTVs, including CTVn_L1, L2, L3, CTVn_IMN, CTVn_L4, and CTVn_SCL RTOG, the mean DSCs were mostly higher than 0.70. The mean 95% HD ranged from 5.50 to 10.93 mm for CTVs. The mean DSCs and 95% HDs did not show a large difference between the contrast-enhanced CT test datasets and non-contrast CT test datasets.

**Table 1:** Comparison of deep learning auto-segmentation and manual contours of organs-at-risk and target volumes using test dataset #1

	Total (n = 19)				Contrast (n = 10)				Non-contrast (n = 9)			
	DSC	STD	95% HD (mm)	STD (mm)	DSC	STD	95% HD (mm)	STD (mm)	DSC	STD	95% HD (mm)	STD (mm)
Organs-at-risk												
Heart	0.95	0.02	4.56	2.33	0.96	0.01	3.83	2.80	0.94	0.02	5.36	1.27
Rt Lung	0.98	0.01	3.61	2.15	0.98	0.00	4.64	2.46	0.97	0.01	2.46	0.69
Lt Lung	0.97	0.01	2.82	0.71	0.97	0.01	3.04	0.76	0.97	0.02	2.59	0.55
Thyroid	0.89	0.05	1.88	0.90	0.90	0.04	1.55	0.65	0.88	0.05	2.25	0.99
Esophagus	0.84	0.06	2.87	1.49	0.85	0.05	2.47	0.91	0.83	0.07	3.31	1.85
Spinal cord	0.82	0.10	2.98	3.10	0.87	0.07	1.58	0.74	0.76	0.10	4.54	3.89
Target												
CTVp_breast	0.94	0.04	5.50	3.17	0.94	0.04	5.13	2.74	0.94	0.04	5.91	3.55
CTVn_L1	0.74	0.08	10.93	6.27	0.71	0.09	13.51	7.10	0.78	0.05	8.07	3.40
CTVn_L2	0.80	0.07	6.36	2.52	0.79	0.07	6.71	2.40	0.81	0.06	5.98	2.60
CTVn_L3	0.64	0.13	7.99	3.81	0.66	0.10	6.97	2.87	0.62	0.16	9.11	4.37
CTVn_IMN	0.72	0.09	5.75	3.36	0.67	0.09	7.53	3.71	0.77	0.07	3.77	1.00
CTVn_L4	0.74	0.12	6.04	6.12	0.67	0.12	8.37	7.61	0.80	0.09	3.45	1.41
CTVn_SCL RTOG	0.78	0.08	6.95	2.89	0.76	0.08	7.85	3.20	0.80	0.08	5.95	2.09

### Dosimetric analysis

**Fig. 3:**
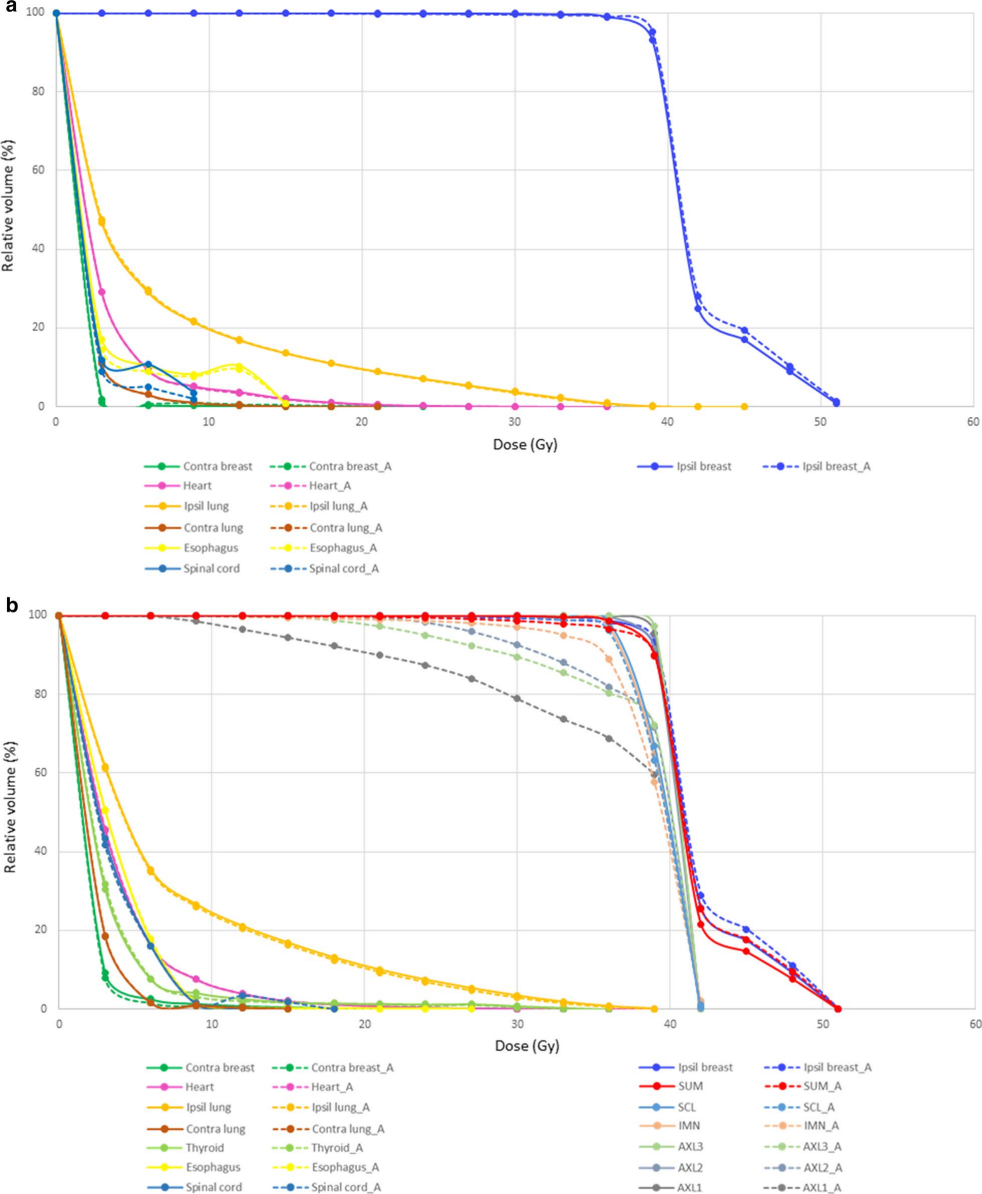
Comparison of dose-volume histograms with average dosimetric values of manual contours (solid line) and auto-segmentation contours (dotted line) for patients who received whole breast RT only(**a**) or that with regional node irradiation (**b**). (Abbreviations: SCL, supraclavicular lymph nodes; IMN, internal mammary lymph nodes; AXL3, axillary lymph node level 3; AXL2, axillary lymph node level 2; AXL1, axillary lymph node level 1)

Figures 3a and b show dose-volume histograms with average dosimetric values for patients who received WB RT only or that with regional node irradiation, respectively. The increase at the end for the ipsilateral breast contour line in the dose-volume histograms is due to the initial RT plan that included a simultaneous integrated boost for the tumor bed. As shown in Fig. 3a, most manual and auto-segmented contours were similar, except for a minor difference for the spinal cord. The difference in the delineated spinal cord volume (average absolute difference of 7.24 ± 9.07cc) may have affected the results. Figure 3b shows that there was a considerable difference in the coverage for regional nodal contours such as axillary lymph node levels 1, 2, 3, and IMN.

In addition, various dosimetric parameters for OARs—such as heart, lung, esophagus, and spinal cord—were analyzed, as shown in Table 2. The mean absolute differences for all parameters were minimal, showing the efficacy of auto-segmented contours.

**Table 2:** Dosimetric outcomes for maual and auto-segmented contours for test dataset #2 (n = 42)

	Manual		Autocontour		Absolute difference	
	Mean	STD	Mean	STD	Mean	STD
Heart						
Mean (Gy)	3.27	1.10	3.26	1.10	0.08	0.07
D_0.03cc_ (Gy)	22.72	10.24	21.75	9.68	1.51	1.76
V_5Gy_ (cc)	16.08	10.23	16.13	10.39	0.73	0.75
Lung						
Ipsilateral lung mean (Gy)	6.87	0.97	6.82	0.97	0.11	0.18
Ipsilateral lung V_20Gy_ (%)	10.08	2.59	9.82	2.68	0.36	0.35
Ipsilateral lung V_5Gy_ (%)	35.25	5.15	35.46	5.32	0.67	0.98
Contralateral lung mean (Gy)	1.83	0.66	1.84	0.67	0.02	0.03
Esophagus						
D_0.03cc_ (Gy)	7.82	5.16	7.56	4.73	0.85	1.61
Spinal cord						
D_1cc_ (Gy)	4.38	2.69	4.37	2.96	0.43	1.08

### Qualitative scoring

**Fig. 4:**
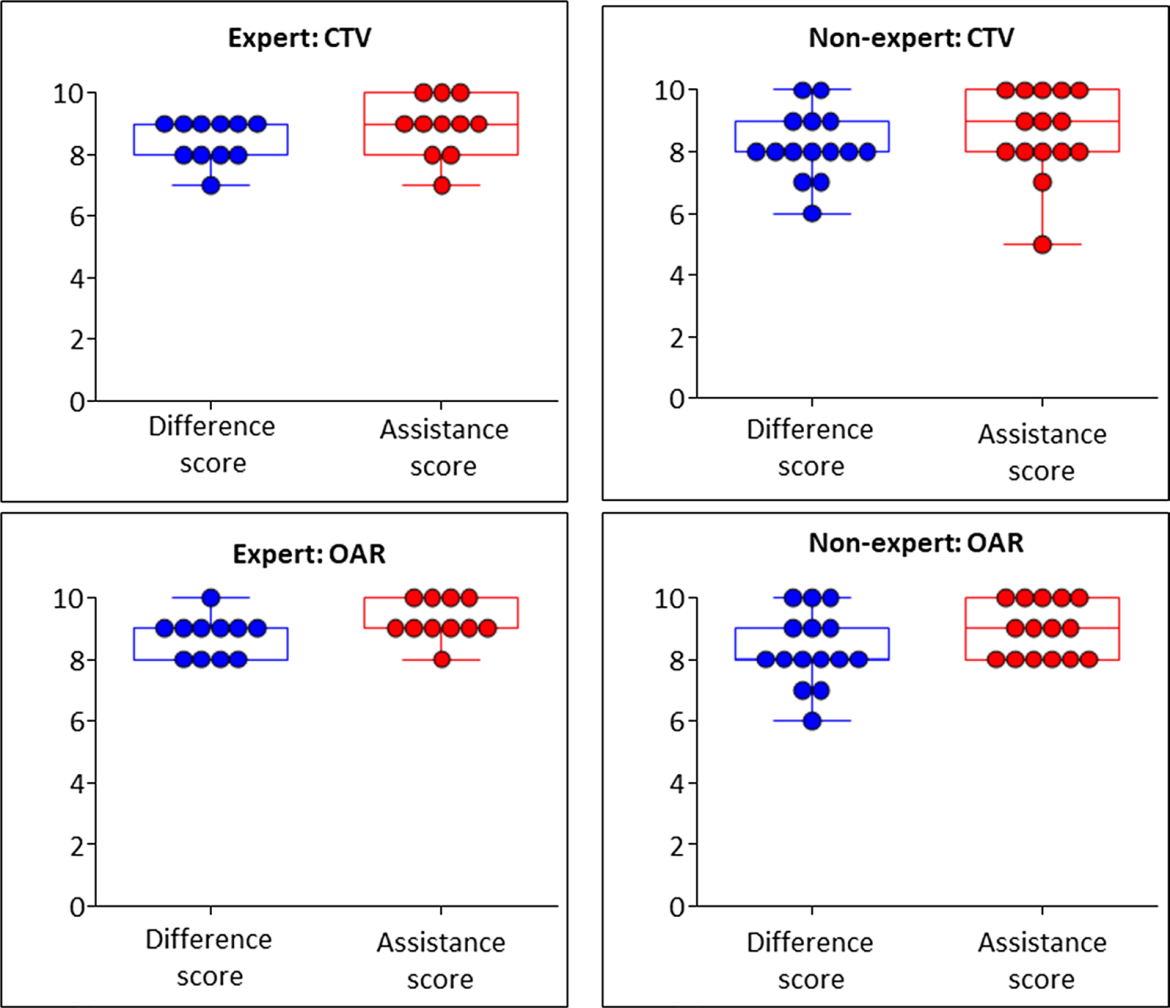
Qualitative scoring by expert panel (n = 11) and non-expert panel (n = 15) for difference and assistance scores

To confirm whether deep learning-based auto-segmentation can practically serve as a useful tool in clinical practice, qualitative scores were also analyzed. Qualitative scoring was performed by both an expert (n = 11) and a non-expert panel (n = 15) for difference and assistance scores, as shown in Fig. 4. For OARs, the median difference score was 9 (range 8–10), and the median assistance score was 9 (range 8–10), in the case of the expert panel. The scores were similar for OARs in the case of the non-expert panel, with a median difference score of 8 (range 6–10) and a median assistance score of 9 (range 8–10). For the CTVs of breasts and regional lymph nodes, the median difference score was 8 (range 7–9), and the median assistance score was 9 (range 7–10), in the case of the expert panel. With regard to the non-expert panel, the median difference score was 8 (range 6–10), and the median assistance score was 9 (range 5–10).

Analyses for inter-observer variability and contouring time are included in Additional file 1.

## Discussion

In this study, we trained a deep learning-based framework to segment 14 CTVs and six OARs in simulation CT images for breast cancer RT. Our findings suggest that the proposed algorithm performed well, exhibiting good agreement with the CTVs and OARs that were manually contoured by clinical experts from both qualitative and quantitative aspects. The dosimetric implications of the auto-contours were also evaluated, and we did not observe any significant difference in dose-volume histograms between the auto-segmented contours and manual contours.

Although AI solutions are best suited to situations in radiology where ground truths are clear, the concept of a ground truth in RT fields is disputable because RT is both a science and an art entailing clinical input and creativity [30]. More specifically, inter-physician variations are present even in contours delineated by board-certified radiation oncologists from the same institution (e.g., variations in the nodal target volumes in our study; Additional file 1). We acknowledge that the generation of the same contours by an AI algorithm under multiple scenarios does not mean that the generated contours are optimal. Considering this, we collected data based on the assumption that the international guidelines are an alternative ground truth [7, 8]. Although the proposed algorithm performed well, a risk exists that its reliability may decrease in some situations [31].

In 2006, Eldesoky et al. first reported the clinical utility of atlas-based auto-segmentation (ABAS) in loco-regional RT for breast cancer using the data of 60 patients, where delineation was performed according to the ESTRO consensus guideline [32]. ABAS showed good agreement in some volumes (e.g., lung, heart, and breast), whereas it showed only modest agreement in other structures or in external datasets. However, research interest shifted to deep learning-based auto-segmentation because ABAS had several limitations; thus, we recently published a study comparing the performance of deep learning-based auto-segmentation with that of two commercially available ABAS systems for breast cancer RT [33]. In this study, the deep learning-based approach showed more consistent and robust performance than ABAS for most structures, and this performance gap increased substantially for soft-tissue-based regions and smaller volumes.

Deep learning-based auto-segmentation has been widely investigated in head & neck, lung, and prostate cancers and has demonstrated clinically relevant impact with regard to saving time and mitigating inter-observer variability [23, 24, 34]. Although several studies have reported the feasibility of the deep learning-based approach for the breast, training and testing has only been performed for ipsilateral breast CTVs [35, 36]. In this study, a satisfactory DSC of 0.94 for CTVp_breast was shown, which is similar to that obtained for other series using the deep learning-based approach. One study using a dataset of 800 patients with a deep learning algorithm (DD-ResNet) showed a mean DSC of 0.91 for the CTVs of both breasts [35]. Furthermore, similar to the study by Eldesoky et al. [32], in which they tested ABAS, we performed training not only for WB CTVs but also for various OARs and other CTVs, including regional lymph nodes in breast cancer patients. The current deep learning-based auto-segmentation model showed higher performance in segmenting various OARs—including heart, lung, thyroid, esophagus, and spinal cord—that were fairly large and well-defined. As for regional lymph nodes, because of the smaller volumes and less well-defined borders, the current model exhibited modest performance. Regarding qualitative scoring, the expert and non-expert panels gave high difference and assistance scores for both the OARs and CTVs.

To date, even with fully validated auto-segmentations, modification or correction by clinical experts is commonly accepted. However, whether modification or correction is essential when auto-contours are utilized for dosimetric analysis has not been well studied. In this study, dosimetric analysis showed that there was a good agreement in dose distribution between manual and auto-segmented contours for OARs. However, as for CTVs, particularly for the axillary lymph node regions and IMN, there were some discrepancies between manual and auto-segmented contours. For OARs, the use of auto-segmented contours for dose–response related studies or for predicting in-clinic normal tissue complication probabilities could be proposed. However, for CTVs, it is apparent that auto-contours in target volumes require significant modification by experts to conform to the corresponding anatomy and to individualize according to tumor and patient information. In the research field, auto-segmented CTVs could be used as a reference point while comparing the target volume delineation of various participants.

In breast cancer trials, variations in target delineation and RT planning have become a prominent issue, particularly in multidisciplinary trials that lack RT quality assurance programs [37]. In a recent audit study across a large network, it was found that nodes were not contoured or the contour quality was inadequate for 18% of patients [38]. In a Korean study that investigated inter-institutional variations in breast intensity-modulated RT (KROG 19–01), there were large heterogeneities in the target volume as well as OARs, producing large variations in mean heart dose and lung V_20Gy_ (up to five times in the same dummy run case). We believe that our auto-segmented contours of CTVs and OARs can play an important role in the breast RT quality assurance process, as illustrated by Chen et al. [36]. Nationwide quality assurance is underway in Korea with our proposed algorithm.

Accurate delineation of all OARs and CTVs is a laborious task; here, auto-segmentation can serve as a useful tool in reducing the workload of physicians. In a previous study on ABAS for loco-regional RT of breast cancer, it was found that ABAS reduced the time required for manual segmentation before correction by 93% and after correction by 32% [32]. This study showed a similar potential, with average times of 39 min and 10 min for manual delineation and deep learning-based auto-segmentation, respectively (Additional file 1). With the assistance of deep learning-based auto-segmentation, radiation oncologists will be able to work more efficiently. Qualitative subjective scoring by the expert and non-expert panels exhibited satisfactory results for both difference and assistance scores, showing that deep learning-based auto-segmentation can serve as a helpful tool in real-world clinics.

This study has several limitations. First, the manual contours used as reference were delineated by a single expert radiation oncologist, which can be considered both as a strength and a limitation. As a single expert delineated all contours, we were able to train the deep learning-based model sufficiently with homogenous data involving fewer patients compared to other studies. However, in the real world, inter-observer variations exist, and there is no 100% gold standard. Thus, we plan to conduct further studies using contours from multiple experts for generalization. However, in a study comparing deep learning-based auto-segmentation of OARs and CTVs based on expert inter-observer variability in RT planning, the accuracy of deep learning-based auto-segmented contours trained using data derived from a single expert was comparable to that of the contours obtained using data with inter-observer variability, thereby showing that the results of the current study with the contours of a single expert radiation oncologist are still meaningful [39].

The second limitation of our study is the number of patients analyzed. However, as mentioned earlier, the DSC for CTVp_breast was better in our study compared to that in other studies. Furthermore, quantitative metrics were not analyzed for test dataset #2 owing to the heterogeneity of the initial contour volumes. Another limitation of our study would be that the position of the patients during CT simulation and treatment may vary among institutions. In addition, different clinical situations, such as post-mastectomy status and reconstructed status, exist for breast cancer patients. In the near future, we plan to validate our deep learning model with data from other institutions. Even with the current results, we are efficiently using auto-segmentation in the clinic by directly sending the CT simulation images to the server after simulation, and the physician uses the auto-segmented contours for RT planning afterwards. However, further research with a larger number of patients is required. Currently, we are engaged in a national multi-center study aimed at validating the results presented herein and leveraging AI techniques to improve the quality of intensity-modulated RT for breast cancer patients (KROG 21–01).

Additionally, it would have been more favorable if coronary vessels were included as an OAR. The importance of cardiac substructures, such as coronary vessels, is being increasingly acknowledged [40]. Currently, the development of a separate deep learning-based auto-segmentation model focused on cardiac substructures, including the right atrium, right ventricle, left atrium, left ventricle, right coronary artery, and left anterior descending artery, is underway.

## Conclusions

This study demonstrated the potential and feasibility of deep learning-based auto-segmentation for breast cancer patients who are receiving RT after BCS. Although deep learning-based auto-segmentation cannot serve as a substitute for the experience of radiation oncologists, it has the potential to serve as a useful tool in assisting them.

## Supplementary Information

**Additional file 1.** Inter-observer variability and contouring time of three radiation oncologists for manual contours of organs-at-risk and target volumes.

**Additional file 2.** Example video of deep learning-based auto-segmentation and manual contours.

## Data Availability

The research data are stored in an institutional repository and will be shared upon reasonable request to the corresponding author.

## Abbreviations

RT: radiotherapy
CT: computed tomography
CTV: clinical target volume
OARs: organs-at-risk
AI: artificial intelligence
BCS: breast-conserving surgery
CNN: convolutional neural network
DSC: dice similarity coefficient
HD: hausdorff distance
ABAS: atlas-based auto-segmentation
