# Supplementary material for: Clinical feasibility of deep learning-based auto-segmentation of target volumes and organs-at-risk in breast cancer patients after breast-conserving surgery
Source: Radiat Oncol. 2021 Feb 25;16:44. doi: 10.1186/s13014-021-01771-z (PMC7905884; doi:10.1186/s13014-021-01771-z)
Supplement: Supplementary file 1 — Additional file 1. Inter-observer variability and contouring time of three radiation oncologists for manual contours of organs-at-risk and target volumes. [file 13014_2021_1771_MOESM1_ESM.docx]

*Inter-observer variability*

Although the differences between the auto-segmented contours and manual contours were assessed quantitatively, in the field of radiation oncology, there is no precise answer or gold standard for CTV and OAR contours. Thus, differences do exist between contours delineated by different radiation oncologists. Supplementary Table 1 shows the inter-observer variability through DSC and 95% HD for OARs and CTVs delineated by three board-certified radiation oncologists for a randomly selected patient. For OAR, only the heart and lungs showed a DSC above 0.80, whereas the other organs showed DSCs lower than 0.80. For CTV, although breast CTV showed an acceptable mean DSC of 0.85, other CTVs such as CTVn_L1, L2, L3, CTVn_IMN, CTVn_L4, and CTVn_SCL showed poor results, with mean DSC ranging from 0.45 to 0.75. For this randomly selected case, the contouring times for the three radiation oncologists were 35, 40, and 42 min, respectively, whereas the time taken to obtain auto-segmented contours was less than 10 min, including the time taken for sending the CT scan to the server and receiving the auto-segmented contours.

| Supplementary Table 1. Inter-observer variability of three radiation oncologists for manual contours of organs-at-risk and target volumes | | | | | |
| --- | --- | --- | --- | --- | --- |
|  |  |  |  |  |  |
|  | DSC | STD |  | 95% HD (mm) | STD (mm) |
| **Organs-at-risk** |  |  |  |  |  |
| Heart | 0.91 | 0.01 |  | 13.00 | 5.10 |
| Rt Lung | 0.99 | 0.00 |  | 2.33 | 0.95 |
| Lt Lung | 0.98 | 0.00 |  | 2.19 | 0.66 |
| Thyroid | 0.72 | 0.07 |  | 5.37 | 1.70 |
| Esophagus | 0.78 | 0.04 |  | 7.08 | 3.52 |
| Spinal cord | 0.69 | 0.09 |  | 72.89 | 49.91 |
| **Target** |  |  |  |  |  |
| CTVp_breast | 0.85 | 0.02 |  | 8.94 | 2.86 |
| CTVn_L1 | 0.69 | 0.04 |  | 13.58 | 3.00 |
| CTVn_L2 | 0.47 | 0.17 |  | 18.74 | 8.15 |
| CTVn_L3 | 0.56 | 0.10 |  | 9.87 | 3.61 |
| CTVn_IMN | 0.53 | 0.09 |  | 35.11 | 17.46 |
| CTVn_L4 | 0.45 | 0.13 |  | 11.82 | 4.88 |
| CTVn_SCL RTOG | 0.75 | 0.03 |  | 6.93 | 0.62 |
